# Supplementary material for: Large-scale dynamics of perceptual decision information across human cortex
Source: Nat Commun. 2020 Oct 9;11:5109. doi: 10.1038/s41467-020-18826-6 (PMC7547662; doi:10.1038/s41467-020-18826-6)
Supplement: Supplementary file 1 — Supplementary Information [file 41467_2020_18826_MOESM1_ESM.pdf]

Supplementary Information: Large-scale Dynamics of Perceptual Decision Information across Human Cortex

Niklas Wilming, Peter R Murphy, Florent Meyniel, Tobias H Donner

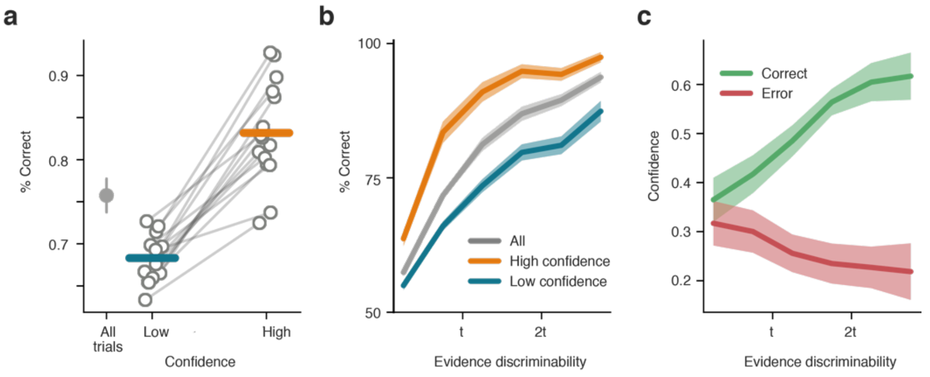

Supplementary Figure 1. Relationships between sensory evidence, choice accuracy, and confidence. a. Choice accuracy, overall and split by participants' confidence reports (high vs. low). Data are represented as group mean (n=15 subjects, horizontal bars, gray dot), ±SEM (gray error bars), and individual participants (gray dots and lines). **b.** Choice accuracy as function of evidence strength in units of threshold (1t = 75% correct; determined by QUEST staircase). Black/gray, overall dependency; colors, dependence split by confidence. **c.** Probability of high confidence report as function of evidence strength, separately for correct and erroneous choices. Taken together, participants' behavioral choices and confidence reports exhibited a lawful dependence on external evidence strength. Specifically, confidence reports revealed three signatures of a measure of statistical decision confidence, defined as the probability of being correct, given the choice made and the evidence (Supplementary References [1–3]): (i) accuracy was larger for high than low confidence (panel **a**); (ii) choice accuracy increased more rapidly as function of evidence strength for high than for low confidence (panel **b**); (iii) probability of high confidence reports increased as function of evidence strength on trials with correct choice, but decreased as function of evidence strength on error trials (panel **c**). Data in b, c are represented as mean (n=15 subjects, lines) and ±SEM (shaded areas). Source data are provided as a Source Data file.

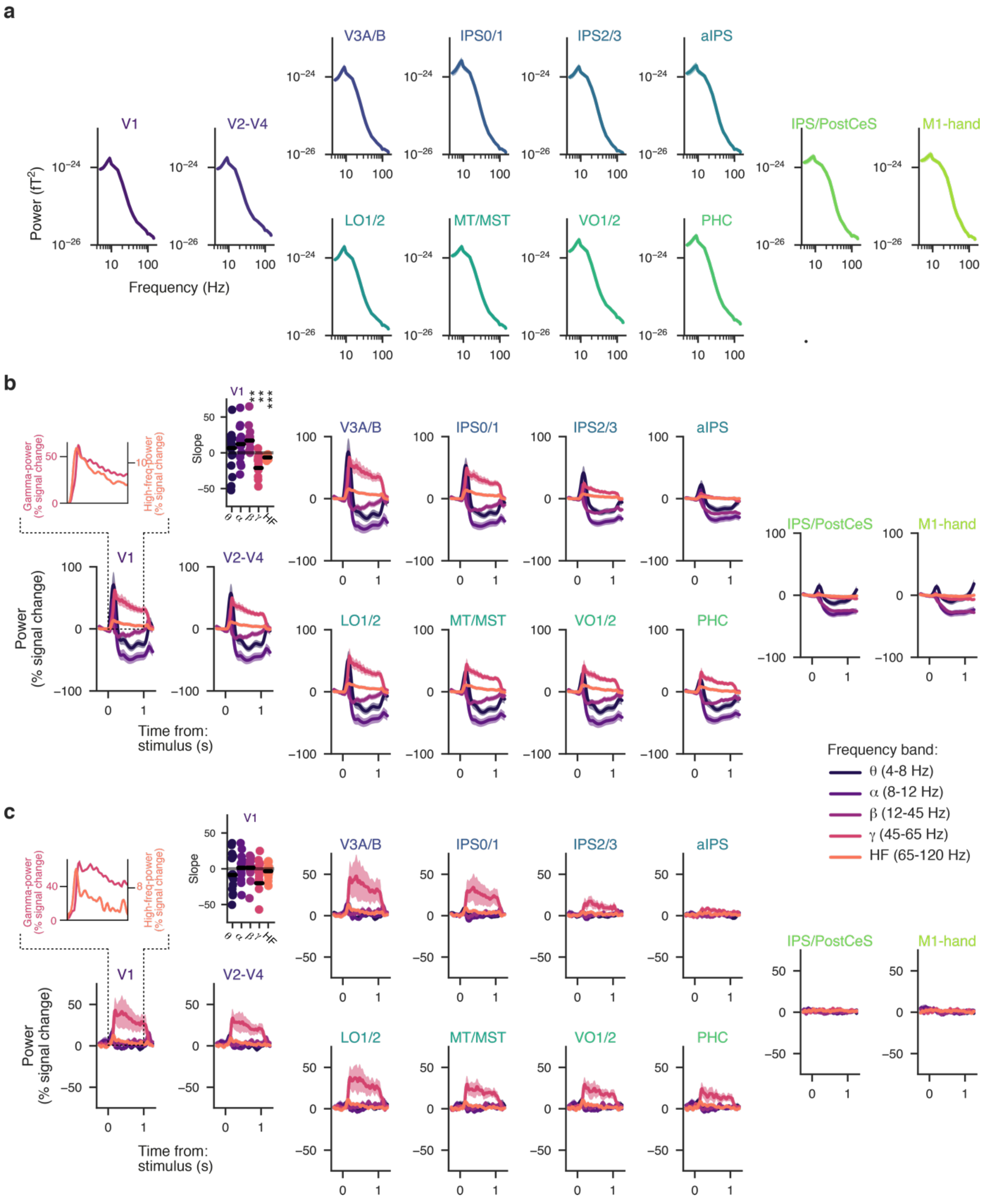

Supplementary Figure 2, Baseline and task-related neural activity in different frequency bands. a. Pre-stimulus baseline power spectra for all cortical regions shown in main Figure 2. Data are represented as group mean (n=15 subjects, horizontal bars, gray dot), ±SEM (gray error bars). **b.** Time course of change of band-limited power relative to baseline during the test stimulus presentation, for same cortical regions as in Figure 2. Frequency bands have been defined so as to span the full range of effects evident in our spectral analysis. Left inset (on top of V1), close-up of V1 power responses in the gamma-band and high-frequency (HF) band. Right inset, slope estimates (linear regression across interval 0.5 to 1s) for V1 power responses in different bands. HF, high-frequency band. Asterisks: **, $p < 0.01$; ***, $p < 0,001$ (two-sided t-tests). β: t(14)=3.9, p=0.002; γ: t(14)=-3.2, p=0.006; HF: t(14)= x-8.6, p=6e^{-7}; **c.** As b, but now for the power response difference between stronger vs. weaker mean test contrast trials. Data in b, c are represented as group mean (n=15 subjects, horizontal bars, gray dot), ±SEM (gray error bars) or individual subjects (dots in inset). Source Data are provided as a Source Data file.

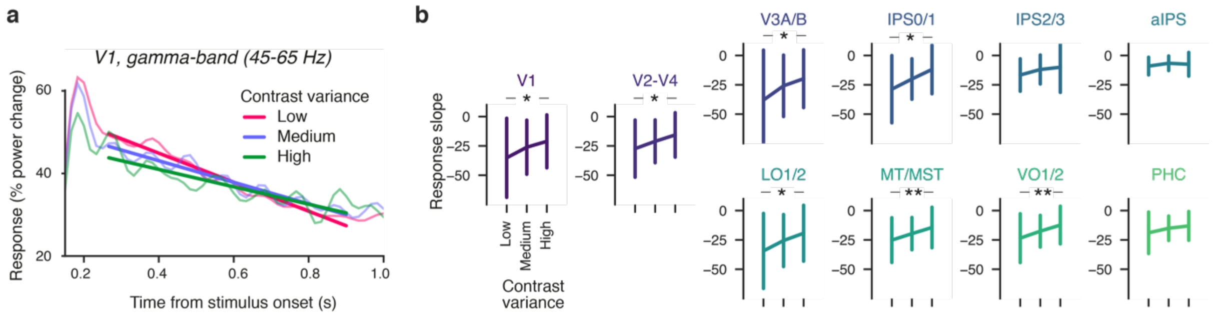

Supplementary Figure 3. V1 gamma-band responses as function of contrast (sample) variance. a. Group average time course of change of V1 gamma-band power relative to baseline during the test stimulus presentation, for three different levels of sample contrast variance during the test (equally spaced from minimum to maximum sample variance). Thick straight lines, fits of linear regressions to group average time course for interval 265 to 900 ms from test stimulus onset, capturing the sustained response component exhibiting the linear down-ramp evident in Supplementary Figure 2b. Data are represented as group mean (n=15 subjects, thin lines). **b.** Group average regression slopes as shown for V1 in a for all visual cortical field maps and as function of sample variance. Data are represented as mean (n=15 subjects, line) and ±SEM (error bars). Asterisks: * = $p<0.05$, ** = $p<0.01$ (repeated measures, 1-way ANOVA for factor sample variance). V1: $F(2,28)=3.92$, $p=0.03$); V2-V4: $F(2,28)=5.3$, $p=0.01$; V3AB: $F(2,28)=3.7$, $p=0.04$; IPS0/1: $F(2,28)=4.0$, $p=0.03$; LO1/2: $F(2,28)=4.9$, $p=0.01$; MT/MST: $F(2,28)=6.2$, $p=0.006$; VO1/2: $F(2,28)=7.1$, $p=0.003$. Source data are provided as a Source Data file.

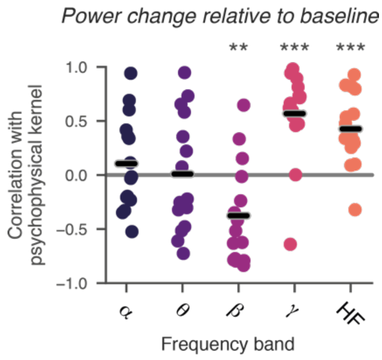

Supplementary Figure 4. Correlation between psychophysical kernels and V1 power dynamics. Temporal correlation between the band-power time courses of V1 from Supplementary Figure 3 and the psychophysical kernels from Figure 1b. Each dot is one participant. Data are represented as mean (n=15 subjects, black horizontal lines) and individual participants (colored dots). Asterisks: **, $p < 0.01$; ***, $p < 0.001$ (two-sided t-tests). β: $t(14)=-3.2$, $p=6.8e^{-3}$; γ: $t(14)=4.5$, $p=4.7e^{-4}$; HF: $t(14)=4.2$, $p=9.4e^{-4}$. These correlations were computed excluding the first two samples so as to eliminate the non-specific onset transient response that spanned all frequency bands (see Supplementary Figure 2b). Correlations were qualitatively identical for gamma and high-frequency (HF) bands when including all samples, but then also significantly positive for theta- and alpha-bands, presumably driven by the onset transient followed by power-suppression. Source data are provided as a Source Data file.

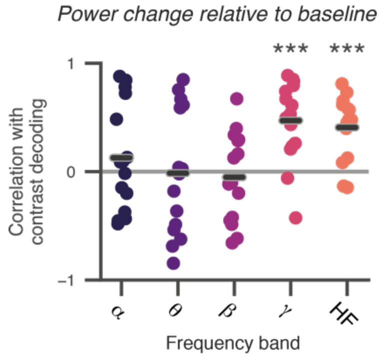

Supplementary Figure 5. Correlation between power dynamics contrast decoding in V1. As Supplementary Figure 3, but now correlating the band-power time courses to the V1 contrast decoding profiles from Figure 3B (left, orange line). Data are represented as mean (n=15 subjects, black horizontal lines) and individual participants (colored dots). Asterisks: ***, $p < 0.001$ (two-sided t-tests). γ: $t(14)=4.6$, $p=4.4e^{-4}$; HF: $t(14)=5.1$, $p=1.7e^{-4}$. The individual temporal profiles of the decoding of momentary contrast samples in V1 (Figure 3B left, orange line) was strongly correlated with the profiles of the gamma-band and high-frequency responses. Thus, at least part of the decay evident in the contrast decoding profiles may be "inherited" from the decay of the underlying power responses (e.g., due to a decrease in signal-to-noise ratio for decoding). Source data are provided as a Source Data file.

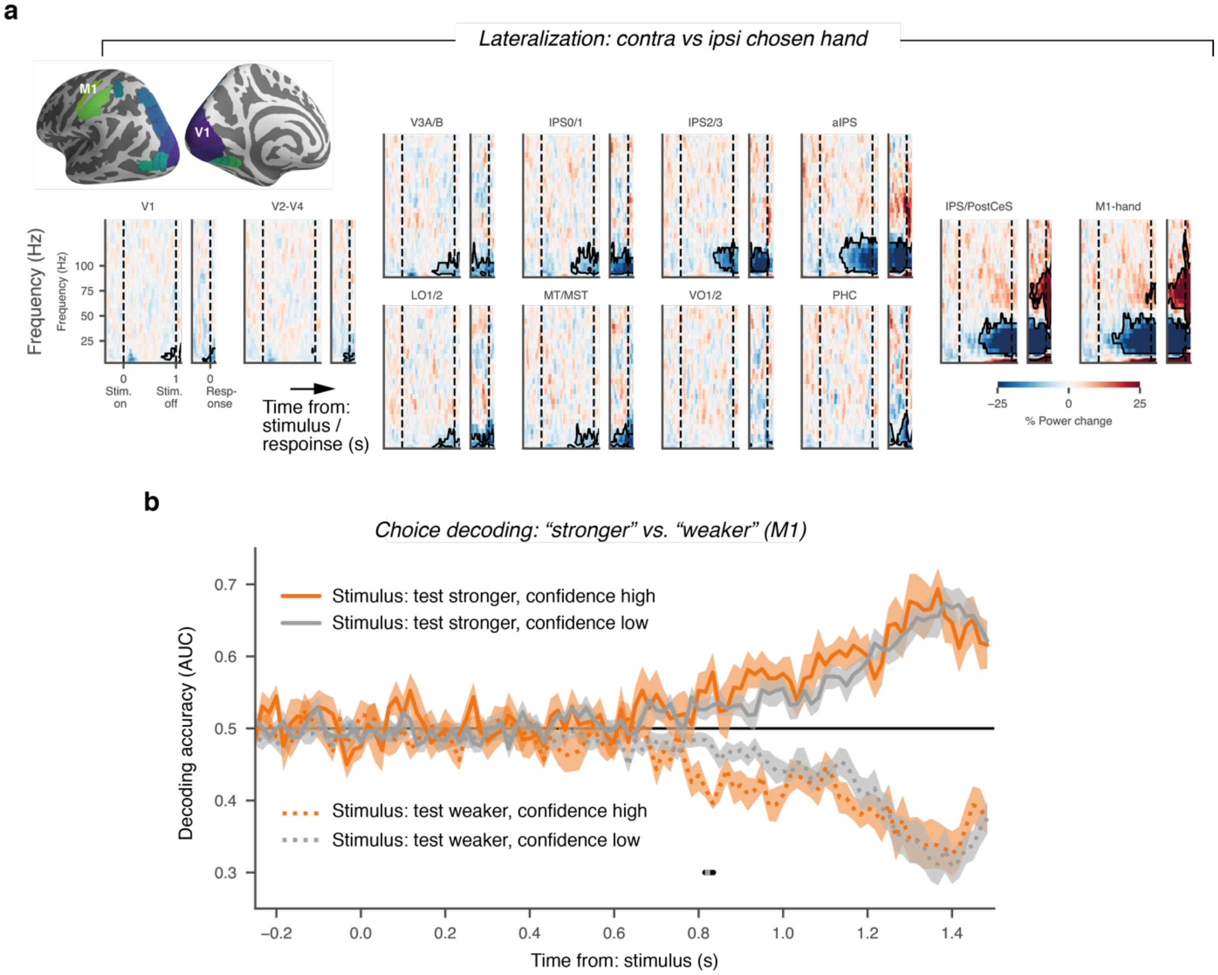

Supplementary Figure 6. Effector choice-related activity lateralization across visuo-motor pathway. a. Time-frequency representations of power lateralization differences "test stronger" vs. "test weaker" choices. We subtracted power values contra-ipsilateral to the hand used for reporting the "test stronger" choice, and then contrasted lateralization values between "test stronger" and "test weaker" choices, separately for the two physical stimulus conditions (see Methods for details). Data are represented as mean (n=15 subjects, color code). Black contour, p<0.05 (cluster-based, two-sided permutation test against 0). Significant choice-related differential activity was evident before the motor response in three frequency bands, and most pronounced in the hand movement-selective ROIs M1-hand, IPS/PostCeS, and aIPS: low-frequency (< 8 Hz, contralateral increase), alpha/beta (8-40 Hz, contralateral suppression), and high gamma-band (50 – 100 Hz, contraleteral increase). Please note that choice-predictive alpha/beta-power lateralization in IPS/PostCeS and M1 became significant about 400 ms after stimulus onset, and then continued to build up throughout decision formation. **b.** Choice decoding, separately per stimulus category, as in Figure 2C, but now also sorted by the reported confidence level (high vs. low). Data are represented as mean (n=15 subjects, lines) and ±SEM (shaded areas). Dashed horizontal bar: p < 0.05 for the Stimulus: test weaker condition (cluster-corrected two-sided t-test of confidence high AUC < confidence low AUC). Only 13 out the 15 participants could be used for this analysis because data needed to be sorted in a total of eight categories for this analysis and not enough trials were available for at least one of them (specifically, the high-confidence error trials) in the remaining participants. Source data are provided as a Source Data file.

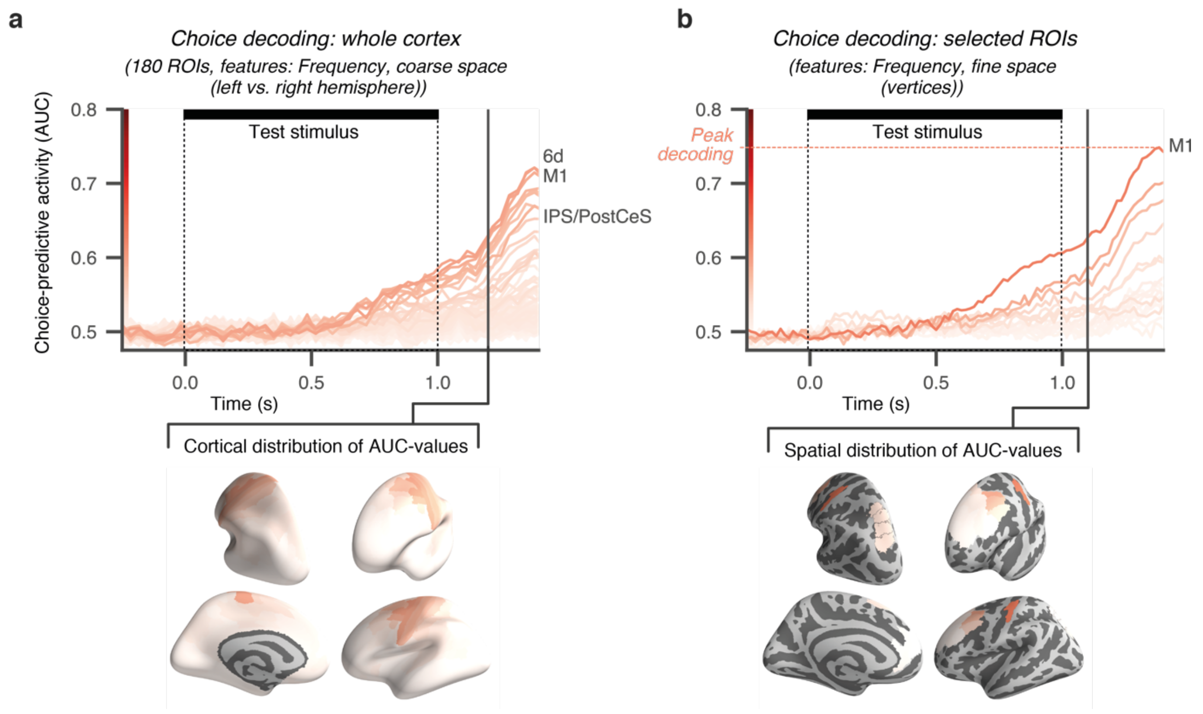

Supplementary Figure 7. Tracking choice-related neural dynamics across cortex. We used two alternatives to the choice-decoding approach used for Figure 2C (approach (i); Methods: *Decoding of choices*). **a.** Approach (ii): Time courses (top) and spatial distribution (bottom) of choice decoding accuracy (AUC, see main text) from 180 ROIs defined by Glasser et al [4]. Choice-decoding was performed separately per stimulus category, as in Figure 2C, and decoding AUC-scores were collapsed across categories, after flipping values about AUC-0.5. ROI-colors encode their AUC-values at t=1.1s (black vertical line, see colorbar next to y-axis). Colors are displayed on inflated cortical surfaces in the bottom row. Strongest decoding performance at t=1.1 was found in three ROIs: dorsal premotor cortex (Brodman area 6d, AUC=0.595), M1 (Brodman area 4; AUC=0.59), and the frontal eye field (FEF; AUC FEF=0.59±0.02). Performance in these ROIs was indistinguishable (repeated measures ANOVA, t=1.1 s, F(2,28)=0.42, p=0.66). All other areas showed decoding performance smaller than M1-hand. **b.** Approach (iii): Time courses (top) and spatial distribution (bottom) of choice decoding accuracy (AUC) from M1-hand (for comparison) and 17 pre-selected ROIs covering dorsolateral prefrontal cortex anterior to premotor cortex. Here, we substantially increased the spatial granularity of the decoding approach by using spectral estimates from each vertex per ROI, and we also included spectral phase (in addition to spectral power) as features for decoding (Methods). Choice-decoding was performed independent of the stimulus category. Again, this showed maximum decoding performance for M1 (AUC=0.63). We found lower decoding performance for all dorsoalateral prefrontal ROIs tested (next best area, Brodman area i6-8 (premotor cortex): AUC=0.59, paired t-test: p=0.002, t=-3.9). Data are represented as mean (n=15 subjects, lines). Source data are provided as a Source Data file.

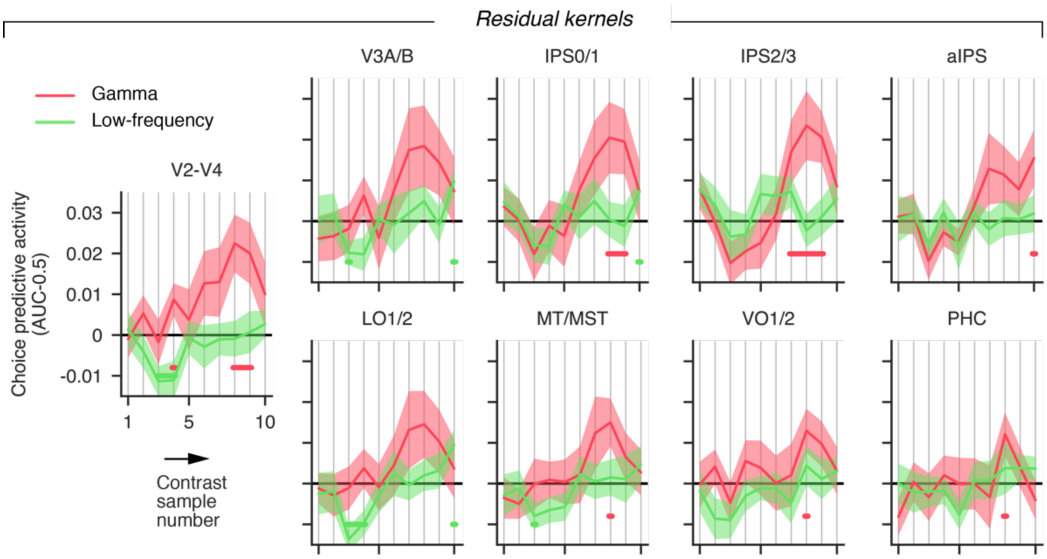

Supplementary Figure 8. Residual alpha- and gamma-kernels across the visual hierarchy. Residual choice-predictive activity (after removing external contrast fluctuations, see main text) across the visual cortical hierarchy. Residual kernels for low and gamma frequency-bands, as in main Figure 4A, but for extrastriate visual cortical field maps. Note the significant choice-predictive residual gamma-band activity on last sample for V3A/B, IPS1/2, and LO1/2. Data are represented as mean (n=15 subjects, lines) and ±SEM (shaded areas). Colored horizontal bar, $p < 0.05$ (uncorrected two-sided t-test of AUC different from 0.5). Source data are provided as a Source Data file.

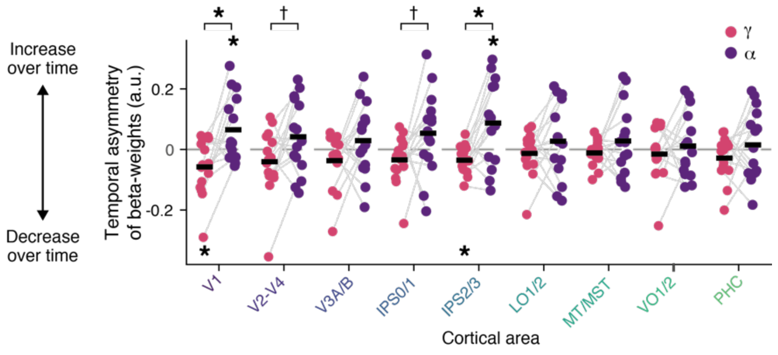

Supplementary Figure 9. Logistic regression of alpha- and gamma-power on behavioral choice. Temporal pattern (first vs. second half of test stimulus interval, within a trial) of gamma- and alpha-band activity on choice, for all visual field maps. Data are represented as mean (n=15 subjects, black horizontal lines) and individual participants (colored dots). Asterisks, crosses: *, $p < 0.05$; †, $p < 0.1$ (two-sided t-tests). V1: γ, $t(14)=-2.39$, $p=0.032$; α, $t(14)=2.31$, $p=0.037$; difference, $t(14)=3.17$, $p=0.007$; V2-V4: γ, $t(14)=-1.42$, $p=0.18$; α, $t(14)=1.32$, $p=0.21$; difference, $t(14)=2.04$, $p=0.061$; IPS0/1: γ, $t(14)=-1.70$, $p=0.11$; α, $t(14)=1.53$, $p=0.15$; difference, $t(14)=1.97$, $p=0.069$; IPS2/3: γ, $t(14)=-3.03$, $p=0.009$; α, $t(14)=2.44$, $p=0.029$; difference, $t(14)=3.47$, $p=0.004$. Source data are provided as a Source Data file.

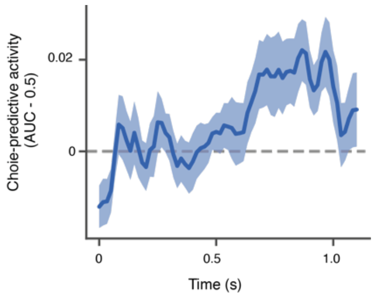

Supplementary Figure 10. Group average V1 low-frequency kernel at higher temporal resolution. For cross-correlations with choice-specific signals in M1, the V1 low-frequency kernels (overall) as in main Figure 4A were recomputed at the same temporal resolution as the M1 choice decoding time courses from Figure 2C. Data are

represented as mean (n=15 subjects, lines) and ±SEM (shaded areas). Source data are provided as a Source Data file.

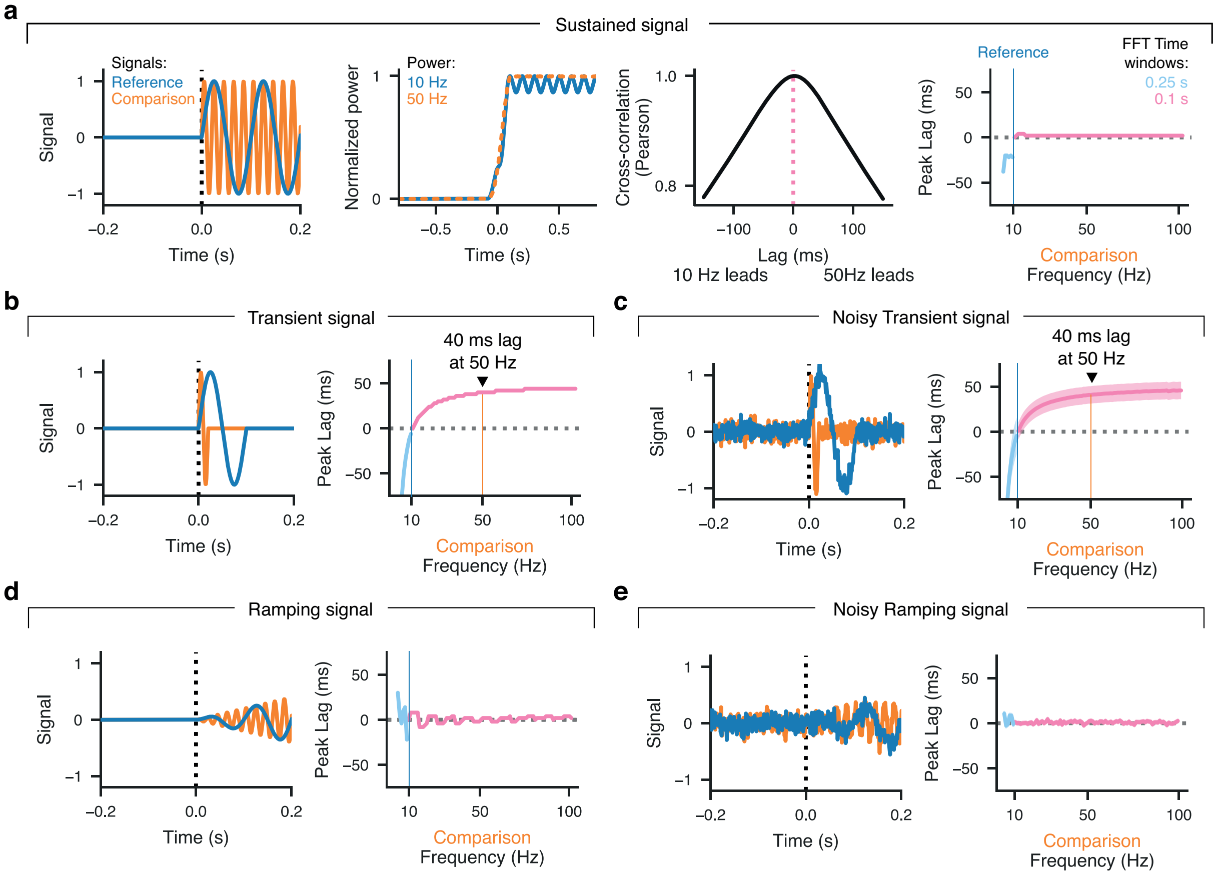

Supplementary Figure 11. Simulation signal-processing induced coupling delays. a. Schematic of simulation procedure (see also Methods). A sustained signal oscillating at 10 Hz ($y_r = \begin{cases} \sin(10*2\pi t)\ if \ \ t \geq 0 \\ 0, \ \text{otherwise} \end{cases}$, 1000 Hz sampling frequency; 2 s duration; "reference" in left panel) starts concurrently with a signal oscillating at 50 Hz ($y_r = \begin{cases} \sin(50*2\pi t)\ if \ \ t \geq 0 \\ 0, \ \text{otherwise} \end{cases}$, "comparison" in left panel). Their respective power change over time is shown in the center left panel (normalized to maximum of one). We evaluated power time courses with the exact same parameters as used for MEG analysis (multi-taper FFT, 100 ms time window for $F \geq 10$ Hz, 250 ms time for $F < 10$ Hz). The cross-correlation between two signals yields a single peak (center right panel). Varying the frequency of the comparison signal shows how the induced peak-lag changes as a function of the frequency difference between both signals (right panel). **b, c**. Same as A, but reference and comparison signals are transient, comprising only a single cycle. Transient signals yield positive lags for larger comparison frequencies and negative lags for smaller comparison frequencies. **b.** Noise-free transient signals. **c.** Transient signals are corrupted by Gaussian noise ($\mu = 0, \sigma = 0.1$). **d, e.** Same as A, but reference and comparison signals are up-ramping linearly. Ramping signals yield positive lags for larger comparison frequencies and negative lags for smaller comparison frequencies **d.** Noise-free ramping signals. **e.** Noise-corrupted ramping signals (same noise as in D). Ramping signals yield results in between those for sustained and transient signals, with small (<25 ms) absolute values of the peak lags. In sum, peak lags in all these simulated scenarios are much smaller than the ones observed in the data (Figure 5). Data are represented as mean (lines) and ±s.d. (shaded areas) of 250 simulation runs.

Supplementary Discussion

Signal processing confounds cannot explain the cross-correlation results

The low-frequency kernels were based on spectral estimates for 0-20 Hz activity and the choice decoder time courses were based on mixtures of different frequencies (1-145 Hz), which were partially computed with different time windows (longer for frequencies < 10 Hz, see section Preprocessing, Spectral analysis, and Source Reconstruction of MEG Data). Another concern about the cross-correlation analysis might be that these signal processing differences might induce artefactual time shifts in the cross-correlation functions (e.g. due to power changes in one band being detected earlier than in the other). This concern might render the peak lags observed in Figure 5 difficult to interpret functionally.

We ruled out this concern by systematically simulating cross-correlations of pairs of oscillatory signals with various different characteristics (transient vs. sustained vs. ramping power increases, pure vs. noisy oscillations) and then analyzed the signals in the exact same way as our actual MEG data (multi-taper sliding window FFT followed by cross-correlation as described above). We observed much higher positive lags in the data than in any of these simulations (Supplementary Figure 11). This renders it highly unlikely that the peak lags reported in Figure 5 were caused by signal processing artefacts.

Specifically, for each scenario, we simulated (250 simulation runs per scenario) a 10 Hz reference signal and a comparisonsignal of varying frequency (e.g., 50 Hz, Supplementary Figure 11a). For sustained power increases of both signals at the same point *t=0* the cross-correlation of power time-course showed no induced time-delays for $F \geq 10$ and negative delays in the order of -25 to -50 ms for $F < 10$ Hz (Supplementary Figure 11a). For two transient signals (single cycle) we found a positive lag of 40 ms (Supplementary Figure 11a, c), because the detection of the power increase differed: The power increase in the 10 Hz signal was maximal when the time-window used for power computation was centered on the peak of the transient 10 Hz signal, i.e. after 50 ms (half of a 10 Hz cycle); the power increase of the 50 Hz cycle was maximal after 10 ms (half of a 50 Hz cycle). This yielded 40 ms as the difference between the two half-cycle durations. Other frequency combinations ($F \geq 10$ Hz) produced a delay of $0.05 - \frac{1}{2F}$ s, which was bounded by 50 ms. Even this lag was substantially shorter than the peak lag observed in the data for early visual cortex (areas V1-V4, Figure 5). Further comparison frequencies < 10 Hz used two different time windows for power computation (as we did for the MEG data analysis) yielded negative delays, opposite to what we found in the data (Figure 5). For the (more realistic) up-ramping signals (linear ramps) we obtained results in between those for sustained and transient signals, with small (<25 ms) absolute values of the peak lags (Supplementary Figure 11d, e).

Comparison of decoding approach with previous approaches for fMRI and MEG data

We here combined atlas-based MEG source reconstruction with a multivariate pattern classification approach that was based on the spectro-spatial patterns of local activity within each region. Current fMRI approaches enable decoding of sensory or cognitive variables from fine-grained multi-voxel patterns in multiple cortical regions [5,6], but they lack the necessary temporal resolution for tracking the dynamics of decision formation. Conversely, E/MEG decoding studies [7–9] provide the critical temporal resolution, but commonly use the whole sensor array as features for decoding, precluding inferences about the information flow between brain regions. Our current approach is situated between these two lines of previous work and thus provides the opportunity to track large-scale information dynamics across cortical areas.

Neural encoding of decision information in motor vs. non-motor formats

Complementary analyses indicated that choice information in anterior intraparietal (IPS/PostCeS) and (pre-) motor cortical regions was primarily contained in large-scale spatial biases (right vs. left hemisphere) with respect to the action (left vs. right hand button press) prepared to report the choice (Supplementary Figure 6a). Indeed, decoding based on the spectral patterns of lateralization yielded very similar results as in Figure 2c (see source data file). We found no robust choice encoding in regions that did not exhibit such large-scale biases. When using finer-grained spatial patterns of both signal phase and amplitude as features for decoding, we did not find strong choice-predictive activity in more anterior regions of prefrontal cortex, even though this yielded higher choice-prediction values for M1 than the coarser-grained approach (Supplementary Figure 7, compare panels b and a, see Methods for all

differences). Previous studies have identified action-independent, choice-predictive signals in human prefrontal cortex during tasks, in which the perceptual choice could not be mapped onto a specific action plan during decision formation [6,10,11]. Our task, however, allowed for such a mapping, mimicking a large body of work in animals [12–15]. The locus and format (action-dependent vs. -independent) of cortical build-up activity during decisions depends on the task context [16]. In our task, the choice-predictive activity may have been exclusively expressed in the format of motor preparatory activity.

Supplementary References

1. Sanders, J. I., Hangya, B. & Kepecs, A. Signatures of a Statistical Computation in the Human Sense of Confidence. *Neuron* **90**, 499–506 (2016).
2. Pouget, A., Drugowitsch, J. & Kepecs, A. Confidence and certainty: distinct probabilistic quantities for different goals. *Nat. Neurosci.* **19**, 366–374 (2016).
3. Urai, A. E., Braun, A. & Donner, T. H. Pupil-linked arousal is driven by decision uncertainty and alters serial choice bias. *Nat. Commun.* **8**, 14637 (2017).
4. Glasser, M. F. *et al.* A multi-modal parcellation of human cerebral cortex. *Nature* **536**, 171–178 (2016).
5. Kamitani, Y. & Tong, F. Decoding the visual and subjective contents of the human brain. *Nat. Neurosci.* **8**, 679–85 (2005).
6. Hebart, M. N., Schriever, Y., Donner, T. H. & Haynes, J.-D. The Relationship between Perceptual Decision Variables and Confidence in the Human Brain. *Cereb. Cortex* **26**, 118–130 (2014).
7. Cichy, R. M., Pantazis, D. & Oliva, A. Resolving human object recognition in space and time. *Nat. Neurosci.* **17**, 1–10 (2014).
8. King, J.-R. & Dehaene, S. Characterizing the dynamics of mental representations: the temporal generalization method. *Trends Cogn. Sci.* **18**, 203–210 (2014).
9. Mostert, P., Kok, P. & de Lange, F. P. Dissociating sensory from decision processes in human perceptual decision making. *Sci. Rep.* **5**, 18253 (2015).
10. Hebart, M. N., Donner, T. H. & Haynes, J.-D. Human visual and parietal cortex encode visual choices independent of motor plans. *NeuroImage* **63**, 1393–1403 (2012).
11. Siegel, M., Buschman, T. J. & Miller, E. K. Cortical information flow during flexible sensorimotor decisions. **348**, 1352–1356 (2015).
12. Gold, J. I. & Shadlen, M. N. The neural basis of decision making. *Annu. Rev. Neurosci.* **30**, 535–74 (2007).
13. Mante, V., Sussillo, D., Shenoy, K. V. & Newsome, W. T. Context-dependent computation by recurrent dynamics in prefrontal cortex. *Nature* **503**, 78–84 (2013).
14. Hanks, T. D. *et al.* Distinct relationships of parietal and prefrontal cortices to evidence accumulation. *Nature* **520**, 220–223 (2015).
15. Pinto, L. *et al.* Task-Dependent Changes in the Large-Scale Dynamics and Necessity of Cortical Regions. *Neuron* **104**, 810-824.e9 (2019).
16. Gold, J. I. & Shadlen, M. N. The Influence of Behavioral Context on the Representation of a Perceptual Decision in Developing Oculomotor Commands. *J. Neurosci.* **23**, 632–651 (2003).
