# Supplementary material for: Large-scale dynamics of perceptual decision information across human cortex
Source: Nat Commun. 2020 Oct 9;11:5109. doi: 10.1038/s41467-020-18826-6 (PMC7547662; doi:10.1038/s41467-020-18826-6)
Supplement: Supplementary file 3 — Reporting Summary [file 41467_2020_18826_MOESM3_ESM.pdf]

nature research

Corresponding author(s): Niklas Wilming, Tobias Donner

Last updated by author(s): Aug 4, 2020

Reporting Summary

Nature Research wishes to improve the reproducibility of the work that we publish. This form provides structure for consistency and transparency in reporting. For further information on Nature Research policies, see Authors & Referees and the Editorial Policy Checklist.

Statistics

For all statistical analyses, confirm that the following items are present in the figure legend, table legend, main text, or Methods section.

n/a	Confirmed	
☐	☒	The exact sample size (n) for each experimental group/condition, given as a discrete number and unit of measurement
☐	☒	A statement on whether measurements were taken from distinct samples or whether the same sample was measured repeatedly
☐	☒	The statistical test(s) used AND whether they are one- or two-sided<br>*Only common tests should be described solely by name; describe more complex techniques in the Methods section.*
☐	☒	A description of all covariates tested
☐	☒	A description of any assumptions or corrections, such as tests of normality and adjustment for multiple comparisons
☐	☒	A full description of the statistical parameters including central tendency (e.g. means) or other basic estimates (e.g. regression coefficient) AND variation (e.g. standard deviation) or associated estimates of uncertainty (e.g. confidence intervals)
☐	☒	For null hypothesis testing, the test statistic (e.g. F, t, r) with confidence intervals, effect sizes, degrees of freedom and P value noted<br>*Give P values as exact values whenever suitable.*
☐	☒	For Bayesian analysis, information on the choice of priors and Markov chain Monte Carlo settings
☒	☐	For hierarchical and complex designs, identification of the appropriate level for tests and full reporting of outcomes
☐	☒	Estimates of effect sizes (e.g. Cohen's d, Pearson's r), indicating how they were calculated

Our web collection on statistics for biologists contains articles on many of the points above.

Software and code

Policy information about availability of computer code

Data collection	MEG and eye-tracking data were collected using the acquisition software developed by the system manufacturer (MEG: CTF Systems Inc., v. 5.4.2); Eye-tracking/pupil: SR Research, v. 4.594). Behavioral data were collected using Matlab version 2016a, using stimulus presentation functions from Psychtoolbox 3.
Data analysis	Custom Python 3 (v. 3.6) code was used for the majority of analyses. MRI-informed source localization of MEG data was carried out with a combination of FreeSurfer (v. dev5-20161028), the MNE toolbox (v. 0.19.dev0) and the FieldTrip toolbox (v. 20170914). Decoding analyses were caried out using Scikit Learn (v. 0.20.1). All custom code is publicly available on GitHub (https://github.com/DonnerLab/2020_Large-scale-Dynamics-of-Perceptual-Decision-Information-across-Human-Cortex).

For manuscripts utilizing custom algorithms or software that are central to the research but not yet described in published literature, software must be made available to editors/reviewers. We strongly encourage code deposition in a community repository (e.g. GitHub). See the Nature Research guidelines for submitting code & software for further information.

Data

Policy information about availability of data

All manuscripts must include a data availability statement. This statement should provide the following information, where applicable:

- Accession codes, unique identifiers, or web links for publicly available datasets
- A list of figures that have associated raw data
- A description of any restrictions on data availability

Behavioral data are available at https://doi.org/10.6084/m9.figshare.12783647. Raw MEG recordings (including eye-tracking data) are available at https://doi.org/10.6084/m9.figshare.12759332. Source-reconstructed MEG data are available at https://doi.org/10.6084/m9.figshare.12770366.

Field-specific reporting

Please select the one below that is the best fit for your research. If you are not sure, read the appropriate sections before making your selection.

☒ Life sciences ☐ Behavioural & social sciences ☐ Ecological, evolutionary & environmental sciences

For a reference copy of the document with all sections, see nature.com/documents/nr-reporting-summary-flat.pdf

Life sciences study design

All studies must disclose on these points even when the disclosure is negative.

Sample size	No statistical methods were used to pre-determine sample size but our sample size is equal to or larger than those reported in previous publications for comparable designs. Of particular note, each of our 15 subjects attended at least 5 sessions (1 training, 4 MEG) and we collected a median of 1787 analyzable trials per participant (range=1128–1872) for MEG data analysis (2500 for behavioral analysis), which is unusually large relative to existing studies (e.g. Siegel et a.l, Cereb. Cortex, 2007; Donner et al., Curr. Biol, 2009; Zylberberg et al., Front. Integr. Neurosci., 2012) and facilitates meaningful data analysis at the level of single subjects.
Data exclusions	No subjects were excluded from all analysis. At the single subject level, we used pre-established criteria based on previous studies from our lab to exclude single trials in which MEG or eye-tracking data were contaminated by well-known artifacts (e.g. by blinks or saccades during the stimulus sequence). For one analysis (Figure S6B), two subjects had not enough trials in some conditions. This is clearly described in the manuscript.
Replication	We did not design our study to replicate the numerous novel effects that we report in our manuscript. However, we are currently running a replication of this study in a larger subject group with additional pharmacological interventions. Results of this replication will be published independent of replication outcome (failed or succesfull).
Randomization	Subjects were all allocated into one group for analysis.
Blinding	Subjects were all allocated to one group in our study and, as such, blinding was not relevant.

Reporting for specific materials, systems and methods

We require information from authors about some types of materials, experimental systems and methods used in many studies. Here, indicate whether each material, system or method listed is relevant to your study. If you are not sure if a list item applies to your research, read the appropriate section before selecting a response.

Materials & experimental systems

n/a	Involved in the study	
☒	☐	Antibodies
☒	☐	Eukaryotic cell lines
☒	☐	Palaeontology
☒	☐	Animals and other organisms
☐	☒	Human research participants
☒	☐	Clinical data

Methods

n/a	Involved in the study	
☒	☐	ChIP-seq
☒	☐	Flow cytometry
☒	☐	MRI-based neuroimaging

Human research participants

Policy information about studies involving human research participants

Population characteristics	Fifteen participants; 8 females, 7 males; age range 21-34. All had normal or corrected-to-normal vision and no history of psychiatric or neurological diagnosis.
Recruitment	All participants were recruited from a large database maintained by the Department of Neurophysiology and Pathophysiology at the University Medical Center Hamburg-Eppendorf. All participants received the following remuneration for their participation: 10 €/h of testing plus a study completion bonus of 40 €. A possible selection bias is that the participant database contains mostly students, which study medicine at the University Medical Center Hamburg-Eppendorf, thereby not forming a representative sample of the general population. This study investigates low-level perceptual decision processes, which we believe to be largely unaffected by this possible selection bias.
Ethics oversight	The experiment was approved by the ethics committee of the Hamburg Medical Association. All participants provided written informed consent.

Note that full information on the approval of the study protocol must also be provided in the manuscript.
